# Mitochondrial deficits and abnormal mitochondrial retrograde axonal transport play a role in the pathogenesis of mutant Hsp27-induced Charcot Marie Tooth Disease

**DOI:** 10.1093/hmg/ddx216

**Published:** 2017-06-08

**Authors:** Bernadett Kalmar, Amy Innes, Klaus Wanisch, Alicia Koyen Kolaszynska, Amelie Pandraud, Gavin Kelly, Andrey Y. Abramov, Mary M. Reilly, Giampietro Schiavo, Linda Greensmith

**Affiliations:** 1 Sobell Department of Motor Neuroscience and Movement Disorders; 2 MRC Centre for Neuromuscular Diseases; 3 Department of Molecular Neuroscience, UCL Institute of Neurology, Queen Square House, Queen Square, London WC1N 3BG, UK; 4 Bioinformatics and Biostatistics Science Technology Platform, The Francis Crick Institute, London NW1 1AT, UK

## Abstract

Mutations in the small heat shock protein Hsp27, encoded by the HSPB1 gene, have been shown to cause Charcot Marie Tooth Disease type 2 (CMT-2) or distal hereditary motor neuropathy (dHMN). Protein aggregation and axonal transport deficits have been implicated in the disease. In this study, we conducted analysis of bidirectional movements of mitochondria in primary motor neuron axons expressing wild type and mutant Hsp27. We found significantly slower retrograde transport of mitochondria in Ser135Phe, Pro39Leu and Arg140Gly mutant Hsp27 expressing motor neurons than in wild type Hsp27 neurons, although anterograde movement velocities remained normal. Retrograde transport of other important cargoes, such as the p75 neurotrophic factor receptor was minimally altered in mutant Hsp27 neurons, implicating that axonal transport deficits primarily affect mitochondria and the axonal transport machinery itself is less affected. Investigation of mitochondrial function revealed a decrease in mitochondrial membrane potential in mutant Hsp27 expressing motor axons, as well as a reduction in mitochondrial complex 1 activity, increased vulnerability of mitochondria to mitochondrial stressors, leading to elevated superoxide release and reduced mitochondrial glutathione (GSH) levels, although cytosolic GSH remained normal. This mitochondrial redox imbalance in mutant Hsp27 motor neurons is likely to cause low level of oxidative stress, which in turn will contribute to, and indeed may be the underlying cause of the deficits in mitochondrial axonal transport. Together, these findings suggest that the mitochondrial abnormalities in mutant Hsp27-induced neuropathies may be a primary cause of pathology, leading to further deficits in the mitochondrial axonal transport and onset of disease.

## Introduction

Mutations in the small heat shock proteins (Hsps) have been linked to a number of hereditary neuromuscular diseases, with mutations in Hsp22 (encoded by *HSPB8*) and Hsp27 (*HSPB1; Accession No NG_008995*) now known to be causative for Charcot Marie Tooth Disease Type 2 (CMT-2) and distal hereditary motor neuropathy (dHMN) (1–6), with mutations in αB crystalline (*HSPB5*) causative for myopathies (7).

To date, over 80 genes coding for proteins linked to several diverse cellular pathways, ranging from protein translation, axonal cytoskeleton and vesicular trafficking to mitochondrial function (8,9) have been implicated in CMT/dHMN. Mutations in Hsp27 are responsible for approximately 4% of CMT2 cases in Italy (10), whereas a more recent study of 17,000 CMT patients established the overall prevalence of Hsp27 mutations at 0.3% among CMT cases and thus, approximately 1% of CMT2 (11).

Hsp27 is an abundant protein, which plays multiple essential functions in cell maintenance and function. As a non-ATPase chaperone, Hsp27 is an important regulator of protein quality control, by preventing the accumulation of denatured proteins, stabilizing the actin cytoskeleton, inhibiting cellular apoptosis and regulating the intracellular redox state (12,13). Disease-causing mutations are distributed along the entire length of the gene and found in both N- and C-termini as well as within the α-crystallin domain, all causing peripheral nerve disease with similar clinical manifestations (14). Most Hsp27 and Hsp22 mutations identified to date are missense mutations causing a single amino acid change in the Hsp27/Hsp22 genes, respectively, and have a dominant pattern of inheritance (9).

Although Hsp27 mutations were first described over a decade ago (1), the pathomechanism by which they result in peripheral nerve disease remains elusive. Protein aggregation and disruption of the cytoskeletal and axonal architecture were initially implicated in mutant Hsp27 pathogenesis (1,15). The study by Ackerley *et al*. (2006) also raised the possibility that CMT-linked Hsp27 mutations may be associated with disturbed axonal transport, as such a mechanism would explain why most motor predominant peripheral neuropathies are length-dependent, first manifesting in distal limbs, and gradually progressing to more proximal nerves (15). Since most CMT-causing mutations follow an autosomal dominant pattern, it is unlikely that they are mediated by a “loss of function” mechanism. Thus, the normal functions of Hsp27 are thought to be unaffected, although it is still possible that haploinsufficiency, caused by the mutant allele of the gene possessing reduced activity, may cause the disease. For example, it has been shown that disease-causing Hsp27 mutations do not affect the phosphorylation levels of the protein, but cause altered activity and an abnormal association with cytoskeletal components, such as tubulin (16–18).

The development of transgenic mice carrying mutant Hsp27 has allowed detailed investigation of *in vivo* and *in vitro* effects of these mutations, although, intriguingly, mouse models of the disease, sometimes expressing the same mutations, show very different phenotypes and severity or no phenotype at all. However, one mouse model overexpressing mutant Hsp27 has been shown to recapitulate many key symptoms of CMT/dHMN, exhibiting a late onset, mild neuropathy, manifesting in reduced motor and sensory nerve conduction velocity and reduced compound muscle action potential (CMAP) between 6 and 10 months of age (19,20). On the other hand, endogenous expression of the mutant Hsp27 protein does not cause peripheral nerve disease or motor symptoms, highlighting the limitations of mouse models to accurately model human disease (21). The study by d’Ydewalle *et al*. showed that in sensory neurons of mice expressing the Ser135Phe Hsp27 mutation, mitochondrial transport is disturbed *in vitro*, with a reduction in the proportion of mitochondrial movements, and this altered mitochondrial transport is coupled to increased acetylation of tubulin in sciatic nerves and spinal cords of mutant Hsp27 mice (20). Moreover, treatment of these mice with histone deacetylase 6 (HDAC-6) inhibitors restored the deficits in mitochondrial movements and improved the clinical phenotype of the mice (20). Importantly, however, the effects of mutant Hsp27 on other key elements of the axonal transport machinery were not examined in this study and therefore remain unknown, and motor neurons, the primary target of dHMN-causing Hsp27 mutations, were not examined in by d’Ydewalle *et al.* Furthermore, since the transgenic mouse examined by d’Ydewalle was created by overexpressing mutant human Hsp27, it is also difficult to establish whether the observed deficits are directly caused by the Hsp27 mutation or a consequence of overexpression of the mutant protein.

In order to elucidate the cellular pathomechanism of mutant Hsp27, in this study, we used cell lines and primary motor neurons expressing three different Hsp27 mutations: Pro39Leu, Ser135Phe and Arg140Gly (3). We investigated whether Hsp27 mutations sensitize cells to cytotoxic insults linked to the primary roles of Hsp27, such as the regulation of cytoskeletal dynamics and intracellular redox state. In primary motor neurons expressing Hsp27 mutants, we undertook a detailed characterisation of the precise nature of axonal transport deficits by examining: i) the bidirectional transport of mitochondria along motor axons; ii) the proportion of moving mitochondria; iii) the speed of mitochondrial movements; and iv) the frequency of pauses between these movements. As a control, we monitored the axonal dynamics of the p75 neurotrophin receptor (p75NTR), which contributes to the transport of neurotrophins including the brain-derived neutrophic factor (BDNF), towards the cell body (22), a process essential for motor neuron maintenance. Finally, we also undertook a detailed analysis of mitochondrial function in mutant Hsp27-expressing primary motor neurons to determine whether deficits in mitochondrial function underlie the reported defects in mitochondrial transport. Our results indicate that mitochondrial transport abnormalities observed in CMT motor neurons are linked to mitochondrial dysfunction, disturbed redox regulation and increased vulnerability to oxidative stress, a mechanism previously only proposed for CMT causing mutations in mitochondrial proteins (23–27). Our results also indicate that in spite of the genetic heterogeneity of CMT2/dHMN, there may be a convergence towards a critical pathomechanism that might be common across different forms of CMT.

## Results

### Mutant Hsp27 reduces neuronal survival and sensitizes neurons to stress

We first examined the effects of CMT-causing Hsp27 mutations on neuronal survival in the presence and absence of cell stressors in differentiated SH-SY5Y neuroblastoma cells resulted in 22–25% transfection efficiency (Supplementary Material, Fig. S1A), with no significant difference in the transfection efficiency of different genotypes (Supplementary Material, Fig. S1B). Transfected neurons were then differentiated left as untreated or were exposed to a range of cellular stressors including H_2_O_2_, to mimic oxidative stress, cytochalasin D, to disrupt the actin cytoskeleton, or colchicine, that affects the microtubule network.

**Figure 1:**
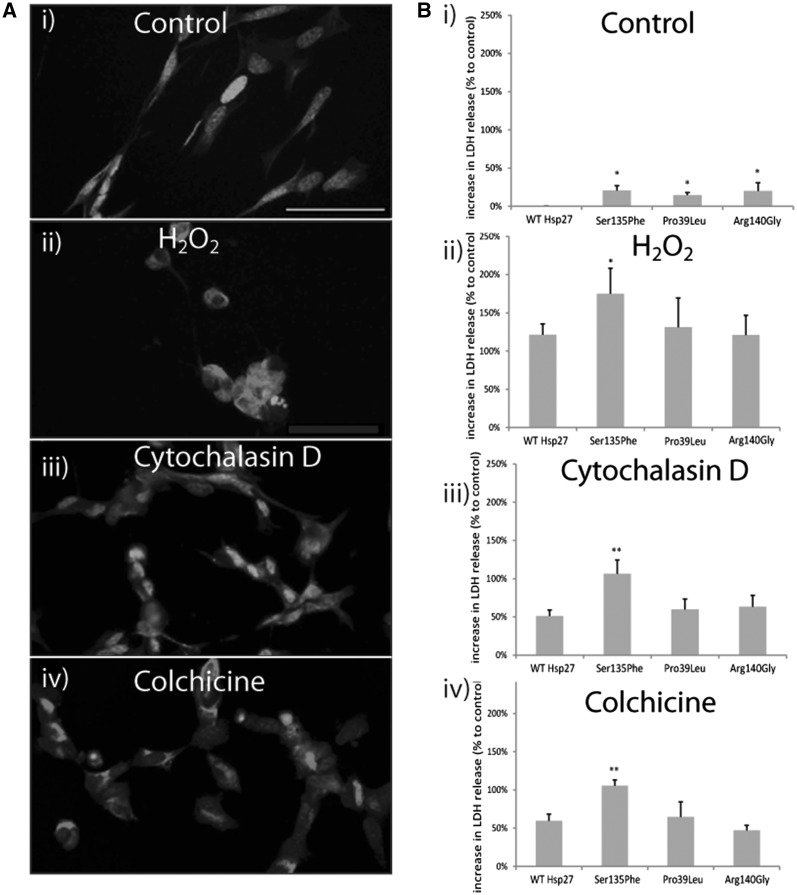
Cell survival in SH-SY5Y cells transfected with wild type (WT) and mutant Hsp27 constructs. (**A**) The images show the morphology of SH-SY5Y cells under i) control conditons and following exposure to ii) H_2_O _2_, iii) cytochalasin D, and iv) colchicine-induced stress. The cells are immunostained for beta III tubulin staining (red) and co-stained for the nuclear marker, DAPI (blue). Scale bar=50µm. (**B**) The bar charts show the extent of cell death assessed using an LDH assay in which LDH release from control and stressed cells was determined 4 days following transfection with WT and mutant Hsp27, and 24-h treatment with the cell stressors. LDH release in experimental conditions is normalized to that of untransfected cells, thus values >0 signify elevated cell death compared to untransfected controls. Error bars= SEM. *P < 0.05; **P < 0.001.

All of these cellular stressors caused significant morphological damage to SH-SY5Y neurons, (Fig. 1A (i-iv)) compared to control, unstressed cells. Even in the absence of stress, we found that Hsp27 mutations caused elevated death in SH-SY5Y cells (Fig. 1B). Thus, in cells transfected with Ser135Phe, Pro39Leu and Arg140Gly mutations cytotoxicity was elevated by 20.8 ± 6.0%, 14.8 ± 3.3%, and 20.1 ± 10.6% (±SEM), respectively (Fig. 1Bi), relative to WT Hsp27. Thus, all the Hsp27 mutations showed a statistically significant increase in cell toxicity under basal conditions (Fig. 1Bi; *P* < 0.05). However, there was no significant difference in the extent of toxicity induced by any of the different Hsp27 mutations.

Treatment with each of the cellular stressors caused significant cell death in SH-SY5Y neurons, compared to control, unstressed cells (Fig. 1Bii-iv). Treatment with H_2_O_2_ (Fig. 1Bii) caused a significant increase (121.5 ± 14.1%, *P* < 0.001, ± = SEM) in cell death in WT Hsp27 cultures compared to unstressed cells. The Ser135Phe expressing cells were significantly more susceptible to H_2_O_2_ than WT Hsp27 expressing neurons, with LDH release levels being 174.8 ± 33.4% greater than unstressed levels (*P* < 0.05). However, neurons expressing Pro39Leu and Arg140Gly Hsp27 were not more vulnerable, with LDH release at 131.2 ± 38.1% and 120.9± 26.0% of control unstressed cells, respectively (Fig. 1Bii).

Cytochalasin D treatment also caused a significant, 51.1 ± 7.8%, (*P* < 0.001) increase in LDH release in WT Hsp27 neurons (Fig. 1Biii). Again, Ser135Phe Hsp27 cells were significantly more vulnerable (106.3 ± 18.1% increase from control (*P* < 0.001)), but the other 2 mutations did not cause a significant increase in cell death (59.8 ± 13.7% in Pro39Leu and 63.4 ± 14.9% in Arg140Gly; Fig. 1 Biii).

Colchicine treatment (Fig. 1Biv) also induced a significant (59.6 ± 8.6%, *P* < 0.001) increase in LDH release in WT Hsp27 neurons (Fig. 1Biv) but again, only Ser135Phe Hsp27 cells were significantly more vulnerable (105.5 ± 7.4%, *P* < 0.001 in Ser135Phe cells; 64.8 ± 19.4% in Pro39Leu and 66.7 ± 16.0% in Arg140Gly above control).

Thus, although all three mutations were found to be mildly cytotoxic, the Ser135Phe Hsp27 mutation also increased the vulnerability of cells to all the cellular stressors tested.

### CMT-causing Hsp27 mutations have no effect on the morphology of primary motor neurons

In order to elucidate the pathomechanism of CMT in a more disease relevant *in vitro* model than immortalised cells, we established a cellular model in which primary motor neurons expressed Hsp27 mutations using 3^rd^ generation lentiviral constructs expressing WT Hsp27 and the Ser135Phe, Pro39Leu and Arg140Gly mutations.

Primary motor neurons transduced with WT and the different mutations in Hsp27 had a similar level of transgene expression, with a mean of 4 mRNA copies present per 1000 actin copies (Supplementary Material, Fig. S2A). Thus, the results from the model examined in this study reflect the effects of a very mild overexpression of human WT and mutant Hsp27 genes. At the protein level, cells could be identified using GFP or the V5 tag (Supplementary Material, Fig. S2Bi-ii). Western blot analysis confirmed that V5 was only present in cultures transduced with the lentivirus containing the Hsp27 gene and the V5 fusion tag (Supplementary Material, Fig. S1Bii) and the level of Hsp27 expression was similar between cells infected with WT Hsp27 and all mutant Hsp27 constructs (Supplementary Material, Fig. S2Ci-ii).

**Figure 2:**
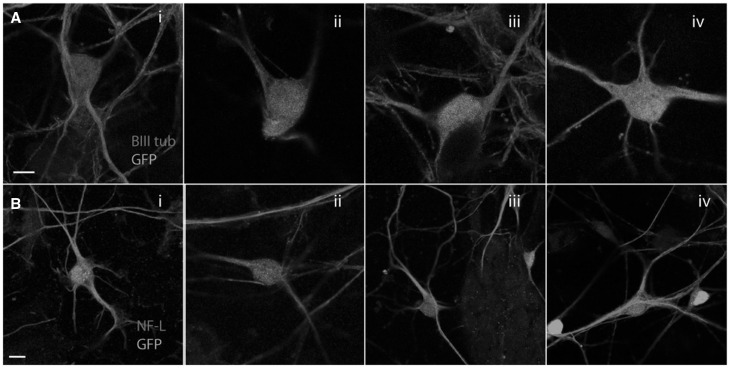
Morphological characterization of primary motor neuron cultures expressing WT and mutant Hsp27. Expression of (**A**) beta III tubulin (red) and (**B**) neurofilament light chain (red) in virally infected motor neurons expressing i) WT Hsp27, or ii) Ser135Phe Hsp27; iii) Pro37Leu Hsp27, or iv) Arg140Gly mutant Hsp27. GFP: green. Scale bars=10 µm.

Morphologically, all mutant Hsp27 expressing neurons appeared normal and neither mutant showed abnormalities in the microtubule or neurofilament networks (Fig. 2) and we did not observe increased toxicity using any of the mutants used (results not shown). The microtubule network appeared unaffected in neurons expressing any of the Hsp27 mutations (Fig. 2Ai-iv) and the expression of NF-L, a cytoskeletal protein proposed to be aggregated by mutant Hsp27, (15) was also normal in all mutant Hsp27 expressing cultures (Fig. 2Bi-iv).

### Primary motor neurons expressing mutant Hsp27 have deficits in mitochondrial axonal transport

Deficits in axonal transport have been proposed to play an important role in the pathogenesis of mutant Hsp27-induced CMT (15,20). However, mutations in Hsp27 result predominantly in a motor phenotype, and previous studies did not examine transport specifically in motor neurons.

**Figure 3:**
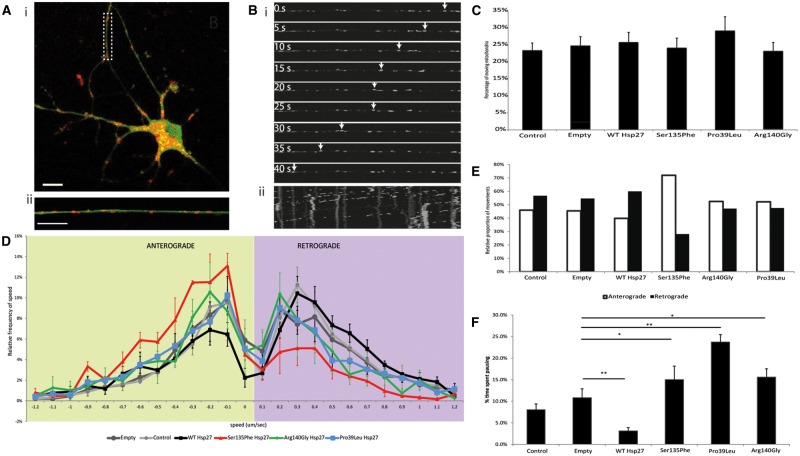
Axonal transport of mitochondria in mutant Hsp27 expressing primary motor neurons. (**A)** i) A typical primary mouse motor neuron expressing GFP (green), indicating transgene expression of WT Hsp27, and co-labelled using Mitotracker (red). ii) A higher power image of an axon of the neuron shown in i) (dotted rectangle). Scale bar = 10µm. (**B**) Live cell tracking of an individual mitochondria within an axonal segment using time lapse microscopy. i) Individual frames of an axon transport movie showing tracking of one depicted mitochondrion (arrows). ii) A kymograph generated using the whole mitochondrial transport movie, with vertical lines indicating stationary mitochondria and some shifted lines indicating movement of mitochondria between frames. (**C**) The proportion of moving mitochondria in motor neuron axons as a percentage of the total number of mitochondria in each experimental condition. (**D**) Relative frequency of the speed of mitochondrial movements. Speed data have been binned and their frequency is shown in each condition in the anterograde (left; green shaded area) and retrograde (right; purple shaded area) direction. (**E**) For each experimental condition, the mean number of movements in the anterograde and retrograde directions is summarised. (**F**) Pause analysis of mitochondrial movements in axons. The percentage of time each mitochondrion spent pausing is calculated for each experimental condition. Error bars= SEM; p > 0.05.

Therefore, we first examined the transport of mitochondria in primary motor neurons. We used time-lapse confocal imaging of live, transduced primary motor neurons and Mitotracker®Red, (Fig. 3A and B). We found that the proportion of mitochondria that moved in motor axons was similar between the different Hsp27 genotypes and untransfected controls, with approximately 25% of the mitochondria in neurites showing movements over the recording period (Fig. 3C). Moving mitochondria were tracked and the speed of the mitochondrial movements recorded (Fig. 3B). Anterograde and retrograde mitochondrial movement speed data was then binned and relative speed frequency distribution graphs generated for each genotype ((28); Fig. 3D).

The relative frequency of mitochondrial movements has a characteristic peak in each direction, which is not significantly different between any of the control cultures used (naïve uninfected and motor neurons infected with either the empty virus or WT Hsp27 (Supplementary Material, Fig. S3). Motor neurons expressing each of the Hsp27 mutants also had normal mitochondrial speed distributions in the anterograde direction, with the frequency distribution curve overlapping with that of controls (Fig. 3D). Retrograde mitochondrial transport was however significantly altered in motor neurons expressing all of the Hsp27 mutants, with slower movements occurring more frequently in Ser135Phe (*P* = 0.00155), Arg140Gly (*P* = 0.0006) and Pro39Leu (*P* = 0.0200) expressing motor neurons (Fig. 3D), causing a shift of the peak towards slower speeds. Analysis of the number of movements in each direction revealed that in motor neurons expressing the Ser135Phe Hsp27 mutation, the ratio between anterograde and retrograde movements shifted to 72% in the anterograde direction and 28% in the anterograde direction, whereas in all other genotypes this ratio remained around 50%. (Fig. 3E). Thus, there was not only a reduction in the characteristic speed of movement of mitochondria, but there was also a characteristic imbalance between the number of anterograde and retrograde movements in these cells, with anterograde movements dominating. In contrast, in controls and motor neurons expressing other Hsp27 mutants, there was an equal balance between the movement of mitochondria in each direction (Fig. 3E).

We also analysed the proportion of time each recorded mitochondrion spent pausing. In this analysis, stationary mitochondria, which have not moved throughout the recording, were not included, and only the length of the pause of otherwise moving mitochondria was assessed. We found that each mutant caused a significant increase in pausing (15.0 ± 3.1%, 23.8 ± 1.7% and 15.6 ± 1.9% in Ser135Phe (*P* < 0.05), Pro39Leu (*P* < 0.001), and Arg140Gly (*P* < 0.05); Fig. 3F), compared to naïve cells and cells expressing empty construct (uninfected controls: 8.1 ± 1.3%; empty virus infected: 10.8 ± 2.0%; Fig. 3F). Interestingly, cells infected with WT Hsp27, mitochondria spent significantly less time pausing (3.1 ± 1.3%, *P* < 0.001) than cells expressing the empty construct (Fig. 3F).

Thus, in mutant Hsp27 expressing motor neurons, mitochondria more frequently moved at lower speeds retrogradely, whereas mitochondrial movement in the anterograde direction was largely normal. Between movements, however, in all Hsp27 mutants examined, mitochondria spent a longer time pausing than in control motor neurons.

### Expression of mutant Hsp27 has marginal effects on retrograde transport of p75NTR in primary motor neurons

**Figure 4:**
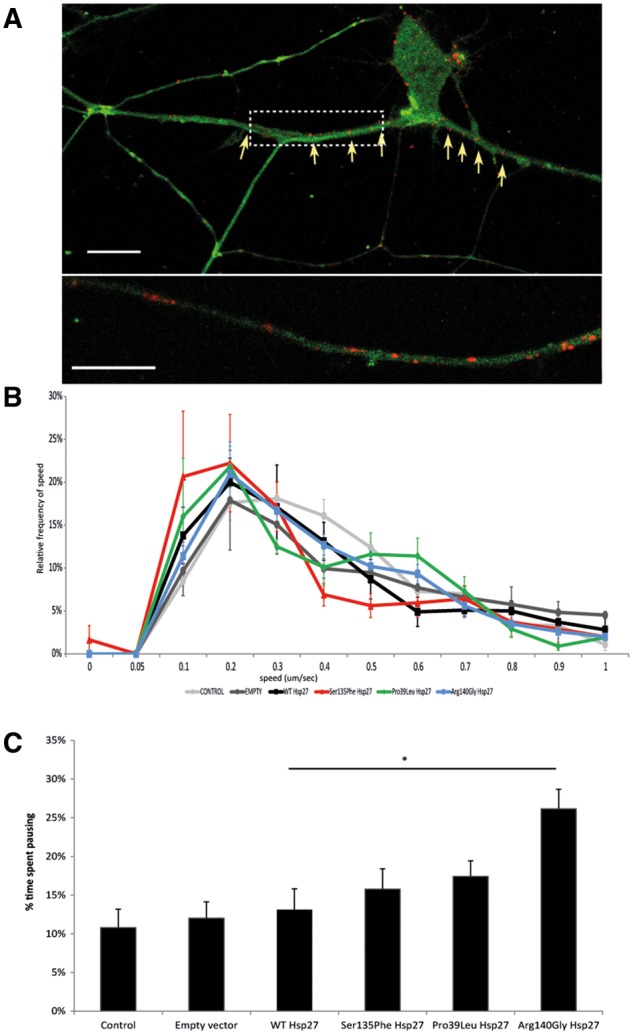
Axonal transport of p75NTR in mutant Hsp27 expressing motor neurons. (**A**) i) A primary mouse motor neuron expressing GFP (green), co- expressed with a WT Hsp27 construct and the intracellular fragment of P75NTR (red). ii) An axonal section selected from the cell in i) (dotted rectangle) is shown at higher magnification. Scale bar=10 µm. (**B**) Relative frequency of the speed of p75NTR movements. Speed data have been binned and their frequency is shown. Error bars =SEM. (**C**) Pause analysis of p75NTR movements in motor axons. The percentage of time each labelled receptor spent pausing is calculated for each experimental condition. Error bars= SEM *P < 0.05; **P < 0.001.

We next investigated the transport of another key cargo, the p75 neurotrophin receptor (p75NTR, Fig. 4A) which is known to bind to neurotrophins together with the family of Trk co-receptors (29), and following internalisation, is retrogradely transported to the cell body through the dynein-mediated transport machinery (30), the same pathway that mitochondria use in retrograde transport.

The speed frequency distribution profile of P75NTR transport was very similar in naïve, untransfected controls, cells transduced with an empty virus and cells transduced with WT Hsp27 expressing virus (Supplementary Material, Fig. S3B). The speed distribution profiles of p75NTR transport of Arg140Gly and Pro39Ley Hsp27 cells were also not different from controls (Fig. 4B; *P* = 0.36 for Arg140Gly and *P* = 0.17 for Pro39Leu Hsp27 mutants, compared to WT Hsp27 control). However, in cells expressing Ser135Phe Hsp27 mutation, there appeared to be a mild deficit in the transport of p75NTR cargoes, with transport in these cells slightly shifted towards slower speeds (*P* = 0.029) Analysis of the time p75NTR cargoes spent pausing between movements revealed that there was no significant difference in neurons expressing Hsp27 mutations compared to controls, apart from one mutant, Arg140Gly Hsp27, which showed a moderate but significant increase in the amount of time p75NTR spent pausing (Fig. 4C; *P* < 0.05).

Thus, in contrast to the deficits in mitochondrial transport observed in mutant Hsp27- expressing motor neurons, the retrograde axonal transport machinery utilized by the p75NTR is largely unaffected, and only the most severe Ser135Phe mutation cause a mild impairment in p75NTR trafficking.

### Impaired mitochondrial function in mutant Hsp27 expressing motor neurons

These findings suggest that the deficits in mitochondrial transport observed in mutant Hsp27-expressing motor neurons may reflect defects in mitochondria. Next we investigated the mitochondrial membrane potential (Δψm) in motor neurons expressing the Ser135Phe Hsp27 mutation, which exhibited the most significant deficits in mitochondrial axonal transport.

**Figure 5:**
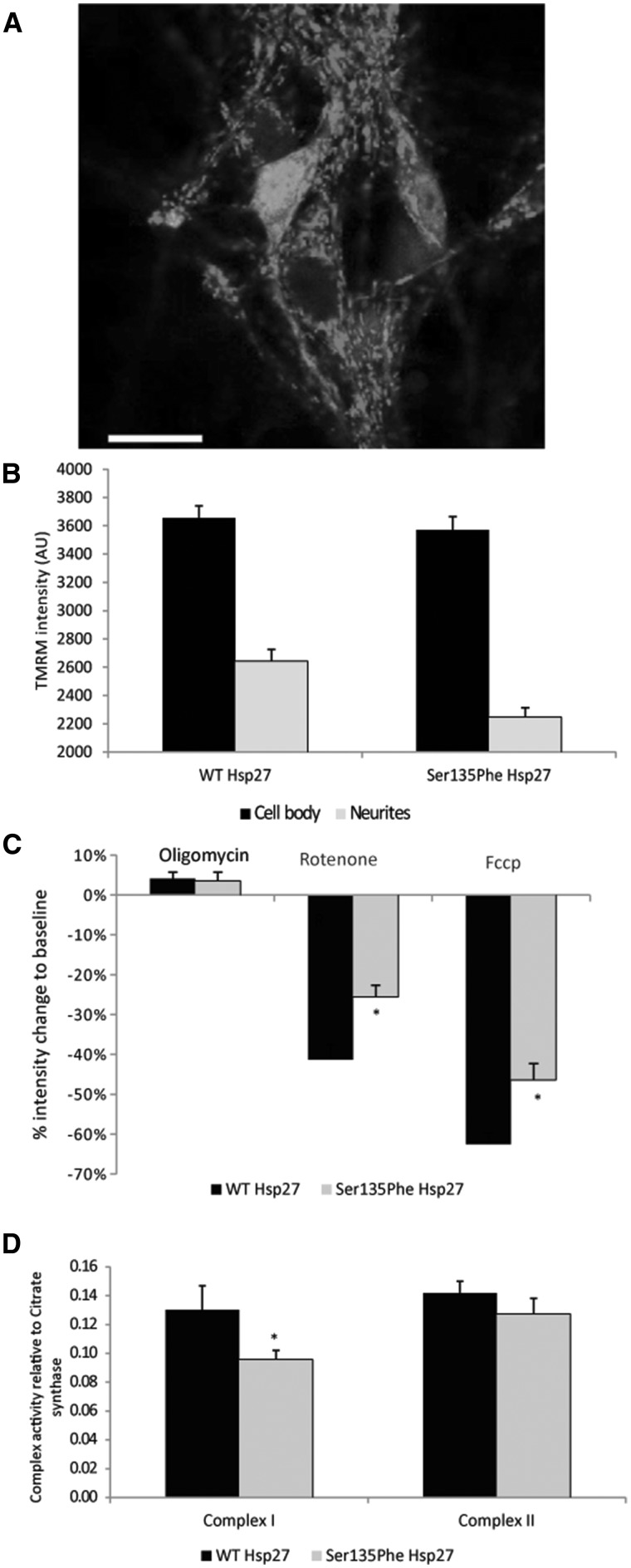
Mitochondrial membrane potential in mutant Hsp27 expressing motor neurons. (**A**) A primary mouse motor neuron expressing WT Hsp27 and GFP, labelled with the potentiometric dye TMRM (red). Scale bar= 20µm. (**B**) TMRM intensity of individual mitochondria measured in WT and Ser135Phe Hsp27 expressing motor neurons. TMRM intensity was measured in both mitochondria of the cell body (black bars) and in neurites (grey bars). (**C**) Changes in TMRM intensity (mitochondrial membrane potential) in response to oligomycin (Complex V blocker); rotenone (Complex II blocker) and FCCP (protonophore). (**D**) Mitochondrial Complex I activity in motor neurons expressing WT and mutant Hsp27. Error bars=SEM *P < 0.05.

Fluorescent intensity of TMRM was measured in motor neurons expressing WT and mutant Hsp27 (Fig. 5A). The Δψ_m_ in neuronal cell bodies of mutant Hsp27 expressing neurons appeared normal, and, as can be seen in Figure 5B, TMRM intensity was not significantly different from that in WT Hsp27 expressing cell bodies (3569 ± 49 AU in mutants, 3655 ± 43 AU in WT controls; *P* = 0.96). In contrast, TMRM intensity in mitochondria located in neurites was significantly lower in mutant Hsp27-expressing motor neurons than in neurites of neurons expressing WT Hsp27 (2247 ± 67 AU in mutants and 2643 ± 82 in WT; *P* < 0.05; Fig. 5B). Thus, whilst mitochondria in the cell body of neurons expressing mutant Hsp27 have a normal Δψ_m_, mitochondria in even relatively proximal neuronal processes (a distance from the cell body of <100 µm) have a markedly decreased Δψ_m_.

Deficits in Δψ_m_ can be due to impairments in mitochondrial complex activities. Therefore, we next treated motor neurons with the following specific mitochondrial inhibitors: i) the complex V inhibitor oligomycin; ii) the complex I blocker rotenone; and iii) the protonophore FCCP. As shown in Figure 5C, treatment with oligomycin caused a similar transient increase in TMRM intensity in mutant and wild type Hsp27 motor neurons (4.1 ± 1.5% and 3.5 ± 2.1% in WT and mutant Hsp27-expressing neurons, respectively). However, the rotenone-induced reduction in TMRM intensity was significantly smaller in mutant Ser135Phe Hsp27 expressing neurons (15.5 ± 2.9%) than in WT Hsp27 expressing neurons (41.3 ± 3.8%; *P* < 0.05). FCCP treatment abolished the proton gradient across the mitochondrial membrane and thus, abolished the Δψ_m_ completely. However, in mutant Hsp27 neurons, FCCP caused a significantly smaller decrease in Δψ_m_, with 46.4 ± 4.0% to baseline, compared to WT Hsp27 expressing cells, in which FCCP causes a 62.4 ± 2.8% loss (*P* < 0.05; Fig. 5C).

Since mutant Hsp27 expressing neurons had a reduced response to the mitochondrial complex I blocker, rotenone, we next examined the mitochondrial complex I and II activities in mutant Hsp27-expressing motor neurons. Ser135Phe mutant Hsp27 cells had indeed lower complex I activity (Fig. 5D; complex I to citrate synthase = 0.095 ±0.008) compared to neurons expressing WT Hsp27 (ratio of complex I to citrate synthase: 0.120 ±0.002; *P *= 0.029). Measurement of complex II activities revealed, however that this complex is unaffected by mutant Hsp27 (complex II activity to citrate synthase 0.142 ±0.006 in WT and 0.133 ±0.010 in Ser135Phe p.0.8; Fig. 5D).

### Mutant Hsp27 neurons are more vulnerable to mitochondrial stressors than WT neurons and display signs of increased oxidative stress

Impairments in mitochondrial complex I are known to cause elevated ROS production, mainly of superoxide anions from mitochondria (31,32). We therefore next examined the effects of the mitochondrial-specific superoxide indicator, MitoSOX and FACS and confocal microscopy on superoxide release in WT and mutant Hsp27 expressing motor neurons.

**Figure 6:**
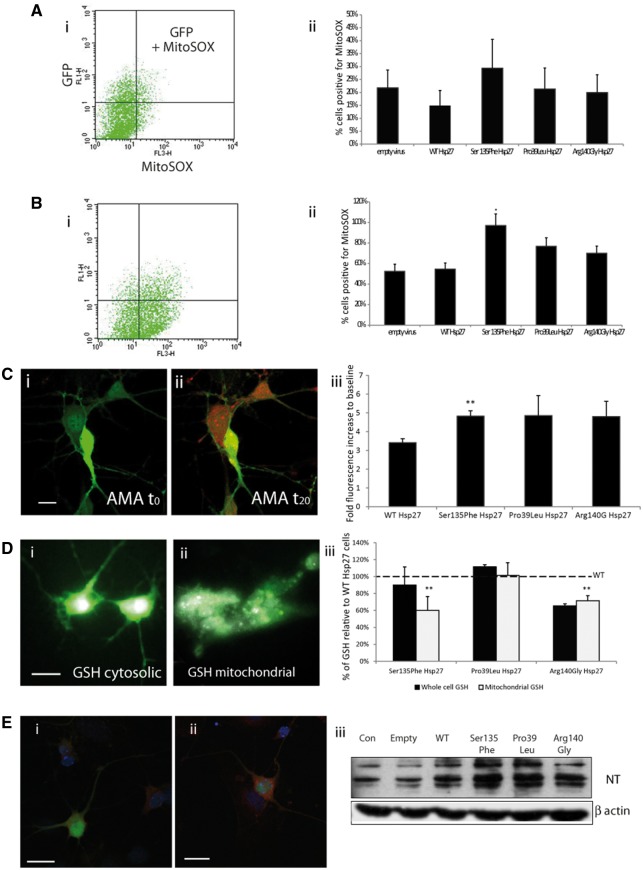
Oxidative stress in mitochondria of mutant Hsp27 motor neurons. To detect mitochondrial superoxide a mitochondria selective superoxide probe (Mitosox) was used, in a system employing FACS analysis (**A,B**) and confocal microscopy (**C**). Ai) A scattergraph showing primary mouse motor neurons expressing WT Hsp27 and GFP under control conditions (FL1H= green channel to detect GFP expressing cells; FL3-H= red channel to detect red mitosox signal). A very small number of cells are above threshold, positive for both GFP and Mitosox. Aii) The bar chart shows the percentage of GFP and Mitosox positive cells expressed within the population of GFP-positive, infected cells, under control, unstimulated conditions. Bi) A scattergraph of the same set of cells as in Ai), following a 20-min incubation with Antimycin A. There is a shift in the number of cells above threshold for both green and red channels, indicating that more GFP positive cells are also positive for Mitosox. The number of GFP and Mitosox positive cells expressed following 20-min incubation with antimycin A is shown in Bii). (C) Confocal images of primary motor neurons expressing WT Hsp27 (GFP) stained for mitosox (red) under (i) control conditions and (ii) following 20-min incubation with Antimycin A. (Scale bars=SEM). (iii) The fluorescent intensity of Mitosox measured before and during the 20-min antimycin A treatment, shown as a fold increase to baseline for each experimental condition. (**D**) Primary motor neurons expressing WT Hsp27 (GFP) and GSH (white) in (i) whole cells and (ii) in mitochondria only. (Scale bars=10µm). (iii) For each set of experiments GSH intensity in whole cells (dark bars) and mitochondria (light bars) was expressed as a percentage of the fluorescence in WT Hsp27 expressing cells. (**E**) Primary motor neurons, infected with an empty vector (i) and (ii) a vector expressing Ser135Phe Hsp27 mutant stained for nitrotyrosine. (Scale bar=20) (iii) Western blot showing nitrotyrosine levels in primary motor neurons infected with lentiviral constructs.

Using FACS analysis, we observed a small, non-significant increase in superoxide signal in all mutant cultures compared to WT Hsp27-expressing cells (Fig. 6Ai-ii). While only 14.9%±5.9% of WT Hsp27-expressing cells were positive for MitoSOX, 29.4 ± 10.0, 21.4 ± 8.1 and 20.0 ± 6.9% of cells expressing the Ser135Phe, Pro39Leu and Arg140Gly Hsp27 mutations, respectively (Fig. 6 Aii; *P* > 0.05).

Next, we examined superoxide release following exposure to the mitochondrial stressor antimycin A (Fig. 6B). The number of superoxide positive cells expressing WT and mutant Hsp27 was determined, which revealed that treatment with antimycin A induced a superoxide signal in significantly more cells expressing the Ser135Phe Hsp27 mutation than in WT Hsp27 expressing cells (54.7 ± 4.3% in WT Hsp27; 97.0 ± 7.4% in Ser135Phe; *P* < 0.05). Similarly, there was an increase in the number of cells positive for superoxide in Pro39Leu and Arg140Gly expressing cells, although this increase did not reach statistical significance (76.0 ± 7.9% in Pro39Leu and 70.1 ± 9.0% in Arg140Gly mutants; Fig. 6Bii).

Since FACS analysis included analysis of all cells in our cultures which expressed both the transgene and GFP, which could include non-neuronal cells as well as neurons, we next used live cell confocal microscopy to investigate the identity of the cells responsible for the increased superoxide release. Although transfected motor neurons had a low MitoSOX fluorescence under baseline conditions (Fig. 6Ci), following treatment with antimycin A, there was a clear increase in the MitoSOX signal, which is indicative of an augmented superoxide release (Fig. 6Cii). Measuring MitoSOX signal intensities following stimulation revealed that in neurons expressing WT Hsp27 there was a 3.41 ±0.21 fold increase in superoxide signal, neurons expressing Ser135Phe had a significantly greater response, with a 4.83 ±0.28 fold increase in the MitoSOX signal (*P* < 0.001; Fig. 6Ciii). The MitoSOX signal also increased in neurons expressing the Pro39Leu and Arg140Gly mutations (4.86 ± 1.0 and 4.81 ± 0.81 fold, respectively), but due to the high variability in responses, this increase was not significant.

### Expression of Hsp27 mutations impairs mitochondrial GSH levels and causes accumulation of oxidised proteins in primary motor neurons

Complex I impairment and increased superoxide production can be the consequence of changes in glutathione (GSH) levels. We therefore next examined cellular and mitochondrial glutathione levels in WT and mutant Hsp27 expressing motor neurons.

Whole cell GSH was measured for individual motor neurons (Fig. 6Di) and following digitonin treatment (Fig. 6Dii) to reveal mitochondrial GSH. Cytosolic GSH levels in the neurons expressing the Hsp27 mutants were not significantly different from that measured in WT Hsp27 neurons (Fig. 6Diii). In contrast, in mutant Hsp27-expressing neurons there was a significant reduction in mitochondrial GSH compared to WT expressing neurons; Ser135Phe only had 60.1 ± 16.4% GSH levels of WT mitochondria (*P* < 0.001), and Arg140Gly expressing neurons had 71.6 ± 5.8% of WT mitochondrial (*P* < 0.001). Interestingly, there was no mitochondrial GSH depletion in the Pro39Leu mutant neurons.

One of the consequences of oxidative stress and impairments in redox homeostasis and excessive superoxide leakage is the appearance of oxidised cellular components, including lipids, DNA and proteins (13,33). In cells expressing mutant Hsp27 we detected an increase in nitrotyrosine, a product of protein oxidation (Fig. 6E). Some mutations, such as the Ser135Phe Hsp27 mutant, showed visibly more nitrotyrosine immunolabelling (Fig. 6Ei-ii), and all Hsp27 mutants were seen to display enhanced nitrosylation in western blots, compared to either uninfected cells or cells infected using the empty vector or WT Hsp27-expressing vector (Fig. 6Eiii).

These results show that the expression of CMT-causing Hsp27 mutations in motor neurons results in a reduction in mitochondrial GSH levels, and this in turn correlates with an increase in the vulnerability of these neurons to oxidative stress and the appearance of damaged, oxidised protein species.

## Discussion

In this study, we investigated the cellular pathomechanisms of peripheral neuropathy caused by missense mutations in the HSPB1 gene encoding for Hsp27. Mutant Hsp27 caused significant cell loss and increased vulnerability to cell stressors, including agents that disrupt the cytoskeletal network. Lentiviral-induced low level expression of human WT and mutant Hsp27 in primary motor neurons causes no morphological abnormalities or protein aggregation. However, there is marked reduction in retrograde transport of mitochondria in primary motor neurons expressing all of the Hsp27 mutations investigated. In contrast, retrograde transport of the neurotrophin receptor p75NTR in primary motor neurons was only detected in motor neurons expressing the Ser135Phe Hsp27 mutant, and even in these cells the deficit in p75NTR transport was mild, with transport in these cells slightly shifted towards slower speeds. Thus, deficits in retrograde transport of p75NTR was only associated with the mutation that induced the most severe deficits in mitochondrial transport, whereas retrograde mitochondrial transport was altered in neurons expressing all of the mutations examined. Furthermore, in neurites of mutant Hsp27 expressing motor neurons, mitochondria display significant functional deficits, including reduced mitochondrial membrane potential, impaired mitochondrial complex I activity, increased superoxide production as well as a significant reduction in mitochondrial GSH levels which correlate with an increased vulnerability to oxidative stress. Indeed, the decrease in mitochondrial GSH levels in Ser135Phe Hsp27 mutant motor neurons is the most significant change observed in mutant Hsp27 expressing motor neurons in this study.

Taken together, our findings suggest that dysregulation of GSH reductase activity by mutant Hsp27 leads to increased vulnerability to oxidative stress, mitochondrial dysfunction and to the deficits in mitochondrial transport described in this study in motor neurons and previously reported in sensory neurons (20,34). Among the Hsp27 mutations tested in this study, the Ser135Phe mutant consistently showed the most profound deficits, with the Arg140Gly and Pro39Leu mutants inducing more varying levels of defects, depending on the assay used. Unfortunately, it is impossible to draw a correlation between these cellular findings and the severity of the clinical phenotype patients with different mutations (1,3,9,10), as the number of patients with each of these mutations is relatively small and even in the same family, the severity of disease and disease onset varies. On the other hand it is notable that the Ser135Phe mutation is one of the most frequently described in European patient populations, which might imply a more complete penetrance for this mutation than the others.”

More than 80 genes have now been described as causative for CMT and dHMN (8,9). All known mutations in members of 3 distinct small Hsps (Hsp22/HSPB8; Hsp27/HSPB1 and αB crystallin/HSPB5) have been associated with diseases of the peripheral neuromuscular system (1–3,5,10,35,36). Interestingly, many mutations in Hsp27, such as Arg136Trp, Ser135Phe and Gly140Phe, and the Lys141Glu and Lys141Asn mutations in Hsp22, are located in a homologous hotspot region of the gene, placed within a conserved β7 strand of the αB-crystallin domain. This domain is crucial for its overall structure and function of the protein (14,37). Most intriguingly, the Arg120Gly mutant of αB-crystallin, that causes a myopathy, is also positioned in the same region, establishing a common causative link between motor dominant CMT/dHMN and muscle disease (14) and in some cases mutations in both Hsps can cause neuropathy and myopathy in the same patient (M. Reilly, personal communication).

Vesicle trafficking and axonal transport abnormalities have been a major focus of research in hereditary peripheral neuropathies, due to the high number of genes involved in either the axonal transport machinery, such as mutations affecting molecular motors, or important elements of the cytoskeleton, such as NF-L and NEFH (38–41), or genes directly responsible for vesicle biogenesis, such as Rab7 (42,43). There is also substantial overlap between the effects on aggregate formation and axonal transport deficits between a number of mutations, including NF-L and Hsp27 (HSPB1), suggesting that a common pathway may be affected by these genes (15,44). Indeed, a number of genes that are causative for hereditary sensory and motor neuropathies are either integral elements of the axonal transport machinery, such as cytoplasmic dynein (dynein heavy chain, DYNC1H1), kinesins (KIF5A and KIF1A) and adaptor molecules dynactin and BICD2 (45–47). CMT causing mutations in cytoskeletal components, such as neurofilaments, also cause abnormal distribution and transport of neurofilaments (48–50). Hsp27 mutations have also been shown to cause disruption in the neurofilament network, leading to the somatic accumulation of neurofilaments, suggesting a role for anterograde axonal transport deficits (15,51), although we did not observe any deficits in anterograde transport in mutant Hsp27 expressing motor neurons in the present study. However, abnormalities in mitochondrial axonal transport have been observed in human induced pluripotent stem cell (iPSC)-derived motor neurons from patients with NF-L and MFN2 mutations (34) as well as mutant Hsp27 expressing sensory neurons (20).

In this study, we conducted an analysis of the effects of Hsp27 mutations on the mitochondrial transport in primary motor neurons, the main neuronal subtype affected in CMT-2/dHMN. Our results showed that although the number of moving mitochondria is not affected by Hsp27 mutations, their speed of *retrograde* movement was significantly slower in Ser135Phe Hsp27-expressing motor neurons. However, the speed of *anterograde* mitochondrial movement was unaffected, leading to an imbalance in the transport of mitochondria in the two directions. These findings contrast with those of d’Ydewalle *et al*. (2011), which reported an almost complete absence of mitochondrial movement in sensory axons obtained from postnatal DRGs rather than motor neurons, which are mainly affected by mutant Hsp27-induced CMT/dHMN. Furthermore, the d’Ydewalle study was focused on the analysis of mutant Hsp27 transgenic mice, in which the mutant protein was significantly overexpressed. It is therefore possible that the mutant protein overload in the DRGs of transgenic mice is responsible for the dramatic reduction in mitochondrial movements reported by these authors. Indeed, a recent study of a knock-in model of mutant Hsp27, in which the mutant protein is expressed at physiological levels, failed to show any motor deficits or any signs of axonal abnormalities (21), leading the authors to conclude that expression of the mutant protein at physiological levels is insufficient to induce CMT-2 pathology within the lifespan of the mouse, at least at the system level. Nevertheless, in the present study, using a primary motor neuron model in which the mutant protein is only mildly overexpressed, detailed, functional analysis of axonal transport was able to detect significant and specific deficits in mitochondrial movement.

Furthermore, we found that retrograde transport of signalling endosomes containing p75NTR was normal in motor neurons expressing two of the Hsp27 mutants, with the Ser135Phe Hsp27 mutation inducing only a minor deficit in retrograde transport of signalling endosomes. These findings suggest that the more severe deficits observed in the retrograde transport of mitochondria are not purely the result of defects in the retrograde transport machinery per se, but may instead be due to the mitochondrial abnormalities induced in neurons by CMT-linked Hsp27 mutations.

Mitochondrial deficits have been previously described in CMT-causing mutations in genes that are directly linked to mitochondrial function, such as Mitofusin 2 (MFN-2) and GDAP1, which are involved in mitochondrial fusion and fission and are known to play a central role in regulating mitochondrial function (52,53). In addition to regulating mitochondrial morphology and mitochondrial complex activity, MFN2 and GDAP1 also play role in regulating mitochondrial movements, with GDAP1 and MFN2 mutations both causing abnormal movement and distribution of mitochondria along the axon, an effect most likely linked to abnormal mitochondrial fusion and fission (24,26,27,54). Other, seemingly non-mitochondrial gene mutations have also been shown to cause mitochondrial deficits, for example NF-L (44), Dynein heavy chain (DYNC1H1; 55) and the muscular dystrophy-related Hsp22 (56). In our study, we observed clear deficits in mitochondrial membrane potential in mutant Hsp27-expressing motor axons, along with reduced mitochondrial complex 1 activity. Interestingly, respiratory chain activity in mitochondria is tightly controlled by mitochondrial fusion and fission and thus, unsurprisingly, neurons expressing GDAP1 also show reduced complex I activity (24,53). Impairments in mitochondrial complex I is the most frequent cause of increased mitochondrial ROS production and in our study, mutant Hsp27-expressing motor neurons not only had a 20% reduction in complex I activity, but were also more prone to increased superoxide release from mitochondria. Reduced complex I activity has previously been linked to, and may be caused by, reduced GSH reductase activity in other neural systems (57–59). Our results in mutant Hsp27 expressing motor neurons support this link, as we observed both a reduction in complex 1 activity as well as reduced GSH reductase activity, which in turn yields the oxidation of thiol groups of NADH ubiquinone oxidoreductase, a redox enzyme of complex I (59). This perturbs the redox balance in mitochondria and promotes the escape of superoxide from the electron transport chain, leading to a persistent, mild oxidative stress, as observed in mutant Hsp27 motor neurons in this study as a small but not significant elevation of superoxide production from our cells in the absence of cellular stress. One of the multiple roles of cellular Hsp27 is linked to its antioxidant activity (60,61), which may be a result of its ability to stimulate the activity of antioxidant enzymes, such as 6-phosphate dehydrogenase (G6PDH) and GSH reductase (13,60,61). This activity of Hsp27 may underlie its protective effects on mitochondrial complex 1 (62). Some members of the small Hsp family, notably Hsp22, which normally are only found in the cytoplasm, can be present in mitochondria under conditions of oxidative stress, although there is limited data on Hsp27 localized to mitochondria under stress (63). Thus, mitochondrial protection by Hsp27 might be indirect, through Hsp22. Whether directly through mitochondrial localization or indirectly through other protein interactions, mutant Hsp27 fails to protect the mitochondria and maintain a healthy redox state in mutant Hsp27 expressing motor neurons and this effect is likely to contribute to CMT disease pathogenesis.

## Conclusions

Taken together, he results of this study suggest that deficits in the retrograde transport of mitochondria in mutant Hsp27 motor neurons are due to mitochondrial impairments caused by reduced mitochondrial complex 1 activity, leading to reduced mitochondrial membrane potential and an increased vulnerability to oxidative stress. Our results also show that mitochondrial GSH reductase activity is reduced in mutant Hsp27-expressing motor neurons, linking the mitochondrial pathology observed in mutant Hsp27 expressing cells to the main canonical functions of Hsp27. Our results therefore indicate that mutant Hsp27-induced CMT/dHMN may be associated with a mitochondrial deficit like other forms of CMT, including mutant GDAP1 and MFN-2–induced CMT. Taken together, our results provide further evidence that mitochondrial dysfunction is an important common pathomechanism in inherited neuropathies. Interventions targeting mitochondrial function are therefore a possible therapeutic target for this group of peripheral neuropathies.

## Materials and Methods

A detailed description of the Methods generating the *in vitro* neuronal cell line and primary motor neuron models as well as standard immunohistochemical and Western blot experiments is found in the Supplementary Materials.

### Assessment of axonal transport

Mitochondrial and p75NTR transport was assessed in mixed mouse motor neurons cultured on MatTek imaging dishes. At 1 DIV, motor neurons were transduced with either WT or mutant (Ser135Phe, Pro39Leu or Arg140Gly) Hsp27-expressing lentiviral constructs. Axonal transport was assessed at 5 to 7DIV by incubating for 30 min with 50 nM Mitotracker®Red (*Life Technologies* M22425) or 2 µg/ml AlexaFluor555-labelled rabbit anti-p75NTR antibody (30) and 50 ng/ml BDNF. Cultures were then washed and maintained in the recording medium (RM; 156 mM NaCl, 10 mM HEPES, 10 mM glucose, 3 mM KCl, 2 mM MgSO_4_, 2 mM CaCl_2_, 1.25 mM KH_2_PO_4_; pH 7.4) for the duration of the experiment.

Imaging was performed using a Zeiss LSM710 confocal microscope equipped with a heated microscope stage, with the temperature in the culture dish at 32–34 °C. Motor neurons were imaged under a 63x objective using the 488 nm excitation wavelength of the Argon laser to visualize GFP expressing virally infected axons, and the 568 nm excitation wavelength of the red laser to visualize either mitochondria or p75NTR. For each axon imaged, the motor neuron cell body was identified in order ensure the direction of transport. A 70 µm segment of the axon was then imaged every 5 s and an image series of 70–120 images was recorded, as described before (28). Carrier tracking on anonymized movies was performed using Volocity software (*Perkin Elmer*). Only moving carriers that could be tracked for at least three time points were analysed and the speed of movement between two consecutive time points was calculated. For each genotype, at least five independent cultures were performed and the total number of carriers for each experimental condition was greater than 50; the number of individual movements analysed ranged between 1,000 and 5,000.

### Live-cell imaging of mitochondrial membrane potential

Cultures transduced with Hsp27 constructs and kept for 5–7 days were incubated with 20 nM TMRM in Recording Medium (RM) for 30 min. Z stack images at 720x720 pixel resolution were captured from each culture condition using a 488 nm line of an Argon laser for imaging GFP and a 543 nm line of a Helium-Neon laser for imaging TMRM intensity. Baseline Δψm was established for individual mitochondria located within cell bodies and neurites separately, using the same set of neurons. The brightest image plane was selected from Z stack images, and then within each motor neuron cell body, the mean TMRM red fluorescence intensity was measured in 5–8 individual mitochondria and the average mitochondrial TMRM intensity was established using the ZEN software. The mean of all mitochondrial measurements for each cell body was considered as the Δψm for each cell. The same method of measurement was applied to mitochondria located in neurites of GFP-expressing neurons.

In order to establish the contribution of mitochondrial complexes I and V to the maintenance of Δψm, a time series experiment was carried out taking single plane images using GFP to identify virally-infected cells and TMRM to assess mitochondrial Δψm every 10 s. Cells were treated with oligomycin (2 μg/ml, *Sigma*), a complex V ATPase inhibitor, to establish whether the mitochondria under investigation were actively synthetizing ATP. After a further 120 s, rotenone, a complex I inhibitor (10 μM, *Sigma*) was added to cells for 5 min. Finally, motor neurons were treated with FCCP (1 μM, *Sigma*), a mitochondrial uncoupler and protonophore, that uncouples oxidation from phosphorylation as it carries hydrogen ions through the membrane and therefore abolishes Δψm, causing a complete depolarization of mitochondria. For each genotype (control, WT or Ser135Phe mutant), data from at least a total of 40 cells were obtained from at least four independent primary cultures.

After background subtraction, the percentage difference from baseline was calculated for each cell after the addition of each mitochondrial inhibitor, giving a measure for the effect of the mitochondrial toxin and the contribution to the total membrane potential of the individual complexes.

### Biochemical assay of mitochondrial complex activities

Primary mixed ventral horn cultures were plated onto 6 well plates at a density of 2x10^5^ cells/well, and transduced with Hsp27 vectors. Cells were harvested at 7DIV, suspended in PBS and stored at -80 °C until further examination. Complex I activity was determined as described by Reed and Ragan (63). Complex II (succinate dehydrogenase uniquinone reductase) activity was determined as described by Birch-Machin *et al*. (64).

Mitochondrial complex activities were normalized to the protein concentration of each sample, determined using a BioRad DC protein assay. Values were also normalized to citrate synthase activity, determined by a method described by Shephard and Hubscher (1969) (65).

### Assessment of oxidative stress in primary motor neurons

Oxidative stress in motor neuron cultures was first assessed using the mitochondrial-specific superoxide probe MitoSOX™ (*Invitrogen*). MitoSOX is a dihydro-ethidium derivative, superoxide-sensitive probe, modified to preferentially localise to mitochondria. Superoxide release was measured by FACS analysis and confocal microscopy (66). See Supplementary Methods for a more detailed description of this measurement.

### Measurement of cellular and mitochondrial glutathione content

To measure cellular glutathione (GSH), motor neurons were incubated in culture media with 50 mM monochlorobimane (MCB; *Molecular Probes*). MCB reacts with the thiol group of GSH to form a fluorescent adduct. Cells were incubated with the dye in RM for a minimum of 40 min in order to reach a steady-state equilibrium. Cells were then washed with RM and fluorescent images acquired using a cooled charge-coupled device (CCD) imaging system using excitation at 380 nm and emission at >400 nm. GFP expressing cells were identified using the 490 nm excitation and emission at >510 nm. Following identification of GFP expressing neurons, 3 consecutive images were taken using the emission filter >400 nm while cells were excited at 380 nm. The RM was then replaced by a “cell internal medium” containing 135 mM KCl, 10 mM NaCl, 0.5 mM KH_2_PO_4_, 1 mM MgCl_2_, 5 mM EGTA, 1.86 mM CaCl_2_ and 40 µM digitonin to enable plasma membrane permeabilisation, and therefore measurement of mitochondrial GSH (67).

In each experiment, whole cell and mitochondrial GSH levels were determined by measuring fluorescence intensity of MCB from images blinded to the experimenter. As fluorescence intensities and instrument settings varied slightly between experiments, the fluorescence of mutant cells was routinely expressed as a percentage of the signal measured in cells expressing WT Hsp27 after unblinding.

### Statistical analysis

LDH data were analysed using the Kruskal-Wallis One Way Analysis of Variance on Ranks. In addition, multilevel mixed model analysis with estimates of fixed effects and multiple comparisons (*SPSS*) was also performed.

Axonal transport data were analysed using a mixed effects linear model. For each mutant genotype, cargo type and direction of travel, we tested the difference between the cargo speed in the mutant genotype and the ensemble of controls using a Wald Test; the fixed effect tested for was the mutant vs control. Random effects were used to compensate for variation between experiments, and for variation between the different genotypes and control. To correct for the multiplicity of tests carried out, we applied Holm’s correction to the *P* values.

All other experiments were analysed using either a non-parametric Mann Whitney U-test, when comparing one single mutant to WT Hsp27 expressing cells, or a Kruskal Wallis one-way analysis, when multiple mutants were used. For all tests, significance was set at *P* < 0.05.

## Supplementary Material

Supplementary Material is available at *HMG* online.

## Supplementary Material

Supplementary Materials

Supplementary Figure 1

Supplementary Figure 2

Supplementary Figure 3
