# Supplementary material for: Mitochondrial deficits and abnormal mitochondrial retrograde axonal transport play a role in the pathogenesis of mutant Hsp27-induced Charcot Marie Tooth Disease
Source: Hum Mol Genet. 2017 Jun 8;26(17):3313–26. doi: 10.1093/hmg/ddx216 (PMC5808738; doi:10.1093/hmg/ddx216)
Supplement: Supplementary Materials [file ddx216_kalmar_hmg_supplementary_materials.docx]

**Supplementary Figure Legends**

**Supplementary Figure 1. Characterization of Hsp27 expression in SH-SY5Y cells**

A) An example of a culture of SH-SY5Y cells transfected with WT Hsp27 and stained for beta III tubulin (green) and V5 (red; identifies cells transfected with Hsp27 fused to a V5 tag). Scale bar=50µm. B) Transfection efficacy in SH-SY5Y cells for WT and mutant Hsp27 plasmid constructs.

**Supplementary Figure 2. Characterization of Hsp27 motor neuron cultures expressing WT and mutant Hsp27.**

A) Quantitative PCR to determine the level of expression of the Hsp27 transgene in primary motor neuron cultures. The number of Hsp27 mRNA copies in each culture was normalised to the number of actin mRNA copies. There is no difference in the number of Hsp27 copies between any of the cultures. B) Primary motor neurons infected with WT Hsp27 lentivirus express a V5 tag fused to Hsp27. i) V5 immunofluorescence (red) appears in GFP (green) expressing cells. ii) Western blot showing the expression of the V5 tag in motor neuron cultures infected with Hsp27 constructs but not in cells infected with the empty construct or the uninfected control.

C) Human Hsp 27 immunofluorescence in primary motor neurons infected with WT Hsp27 lentivirus. i) Hsp27 immunofluorescence for Hsp27 (red) co-expressed with GFP (green). ii) Western blot for human Hsp27. The Hsp27 band only appears in cells infected with Hsp27 constructs. In control, uninfected and empty virus infected cells a weak Hsp25 band appears due to cross reactivity between Hsp27 and Hsp25. Scale bars=10 µm. Error bars=SEM.

**Supplementary Figure 3. Analysis of axonal transport in control cultures**

A) Relative frequency of the speed of mitochondrial movements in naïve, untransduced, neurons, neurons transduced either with empty construct or a viral vector expressing WT Hsp27. Speed data have been binned and their frequency is shown in each condition in the anterograde (left; green shaded area) and retrograde (right; purple shaded area) direction. B) Relative frequency of the speed of P75NTR retrograde transport in naïve, untreated motor neurons, motor neurons transduced with an empty and a WT Hsp27 expressing viral construct.

## Supplementary Materials

## Generation of bacterial plasmids encoding mutant Hsp27

The Hsp27 (HSPB1; Accession No NG_008995) mutations were generated using pcDNA3.1/V5-His TOPO plasmids (Invitrogen, Paisley, UK) containing wild type (WT) Hsp27 (a kind gift of Prof Timmermann, Belgium) as a template for site-directed mutagenesis. Plasmid constructs also expressed a V5 tag as well as GFP. CMT-2 linked Hsp27 mutations were generated using the Quickchange II site-directed mutagenesis kit (Stratagene) using the following primers: Pro39Leu (CTG CCC CGG CTG CTG GAG GAG TGG TCG); Ser135Phe (CAT GGC TAC ATC TTC CGG TGC TTC ACG) and Arg140Gly (CGG TGC TTC ACG GGG AAA TAC ACG C). Individual colonies from One Shot® TOP10 cells transformed with wildtype (WT) and mutant Hsp27 were expanded, plasmid DNA was extracted and sequenced by the Scientific Support Services at the Wolfson Institute for Biomedical Research (London, UK) to confirm the presence of full length HSPB1 gene (primers Forward: ATA GCC GCC TCT TCG ACC AG; Reverse: ACT TGG CGG CAG TCT CAT C). The same protocol and primers were used to confirm the presence of specific mutations. For each construct, four colonies were selected, subcultured and sequenced. Constructs containing the full HSPB1 with the correct mutations were used in further transfection experiments. Bacterial plasmid DNA was purified using the Endofree Plasmid Maxi Kit (Qiagen, Crawley, UK), following the manufacturer’s instructions.

## Generation of lentiviral vectors expressing wild type and mutant Hsp27

**To assess the effects of mutant Hsp27 on motor neurons, we generated lentiviral constructs expressing WT and mutant Hsp27. The pcDNA3.1b bacterial plasmids expressing WT Hsp27 and the Hsp27 mutants Pro39Leu, Ser135Phe and Arg140Gly were used to subclone the relevant *HSPB1* genes into the third generation lentiviral backbone pCDH1-MCS1-EF1-copGFP (*Systems Biosciences, SBI*). Pseudoviral particles expressing WT and mutant Hsp27 were then produced in HEK293T cells using Lipofectamine 2000 (*Invitrogen, Paisley, UK*) by co-transfecting the relevant transfer viral vectors with third generation packaging plasmids expressing the *gag* and *pol* genes (pMDLg/pRRE plasmid) and *rev* gene (pRSV-Rev plasmid). These plasmid also expressed the VSVG envelope gene (pMD2.G plasmid, all plasmids from *Addgene*). Pseudoviral particles were harvested at 48 and 72 hours after transfection and cell supernatants were concentrated by ultracentrifuging at 48 000x*g* for 2.5 hours. The resulting pellets were reconstituted in 200 µl PBS, aliquoted and kept at -80 ˚C until further use. The viral titre for each construct was established by FACS analysis by infecting HEK cells and counting the number of cells expressing GFP 72 hours after transfection. The viral titre for the constructs was 6x10^7^ Transducing Units/ml calculated for HEK293T cells (±10% variability between individual constructs and viral batches).**

**For primary neuronal cultures the viral stock was diluted 1 in 1, 000 and added to mixed ventral horn primary cells plated at a density of 50,000 cells per coverslip.**

## Primary motor neuron cultures

Mixed ventral horn cultures (containing ~50% primary motor neurons), were prepared using a protocol adapted from that described by Camu and Henderson, (1992). Thirteen days (E13) mouse embryos were removed from C57BL/6 mice in accordance with the code of practice for the humane killing of animals under Schedule 1 of the Animals (Scientific Procedures) Act 1986. Embryos were transferred into Hank’s Balanced Saline Solution (HBSS; *Sigma-Aldrich)* supplemented with 2% penicillin/streptomycin. Spinal cords were removed from the embryos, the meninges removed and the ventral horns separated, transferred into HBSS containing 0.025% trypsin solution (type XII-S) (*Sigma)* and incubated at 37˚C for 10 min. Tissues were then gently triturated in L15 medium containing 0.4% bovine serum albumin (BSA) and 0.1 mg/ml DNAse (*Sigma)*. The cell homogenate was then centrifuged through a cushion of 4% BSA for 5 min at 370x g. The pellet was resuspended in complete neurobasal medium (CNB*; GIBCO*) containing 2% B27 supplement *(GIBCO*), 2% horse serum (HS; *PAA*), 0.5 mM L-glutamine *(GIBCO*), 0.05% 2-mercaptoethanol *(GIBCO*), 500 pg/ml Ciliary Neurotrophic Factor (CNTF; *Alomone Labs, Bucks, UK)*, 100 pg/ml Glial Derived Neurotrophic Factor (GDNF; *Alomone)*, 100 pg/ml BDNF (*Alomone*) and 1% penicillin/streptomycin. Cells were then plated either onto 30 mm diameter glass bottom imaging dishes (*MatTek, USA*) at a density of 1x10^5^ cell/dish for imaging experiments, or 13 mm glass coverslips in 24 well plates (5x10^4^ cell/well) for immunohistochemistry and qPCR, or 6 well culturing dishes (2x10^5^ cell/well) for western blot experiments and biochemical assays. All culture surfaces were pre-coated with poly-ornithine (1.5 µg/ml; *Sigma)* and laminin (5 µg/ml; *Sigma).*

## Immunohistochemistry

The cells were fixed using 4% paraformaldehyde in PBS for 15 minutes, then immunostained. The following antibodies were used for immunohistochemistry: goat anti Hsp27 (*Santa Cruz* SC-1049; 1:100), rabbit anti-beta III tubulin (*Covance* 1:500), goat anti-neurofilament light chain (NFL, *Santa Cruz* SC-12966), mouse anti V5 tag (*Sigma* V4014; 1:500) and mouse anti nitrotyrosine (*StressMarq* SMC-154; 1:100). Fluorescently-labelled secondary antibodies were from Thermo Fisher and used at a dilution of 1:1,000.

## Western blot

Primary motor neurons infected with WT and mutant Hsp27 viruses were kept in 6 well dishes for 6 days before they were washed, once in cold PBS then lysed in RIPA buffer containing 50 mM Tris-HCl pH 7.5, 150 mM NaCl, 1% NP40, 0.5% sodium deoxychlorate, 0.5% SDS, 1 mM EDTA and a cocktail of protease inhibitors. Protein concentration was determined in each tissue lysate using a Bio-Rad DC protein assay kit (cat#500-0112), which is based on a Lowry essay method. Each sample was then diluted using 4x Sample Buffer (*Bio-Rad*, cat#161-0747) and RIPA buffer to 1 mg/ml. Lysates were then boiled for 5 min to denature proteins, run on 12% SDS-PAGE gels and then probed for Hsp27 (*Santa Cruz* SC-1049, 1:1,000), V5 (*Sigma* V4014; 1:5,000) and nitrotyrosine (SMC-154; 1:1,000).

**Quantitative PCR**

Primary motor neurons cultured in 24 well plates, infected at 1 DIV with lentiviruses encoding WT and mutant Hsp27 were washed twice in PBS then lysed in 800 µl of Trizol (*Quiagen*), followed by addition of 200 µl chloroform. Samples were then centrifuged at 12,000g for 15 min and the supernatant phase then mixed with 500 µl isopropanol and 2 µl Pette Paint (*Novagen*). Samples were centrifuged again at 12,000 g for 25 min. The isopropanol supernatant was then discarded and the pellet washed in 70% ethanol. Following a short 5 min centrifugation at 7500g, the ethanol supernatant was removed, the pellet air dried and reconstituted in 13 µl RNAse free water and RNA concentration determined using a NanoDrop ND1000 spectrophotometer.

For reverse transcription, Superscript III reverse transcriptase kit (*Invitrogen*) was used and 0.5 µg of RNA, 0.5 mM dNTP (*Sigma*), 2.5 µM oligo(dT) (15 bp, *Invitrogen*) and 2.5 µM random hexamers (*Invitrogen*) were first incubated at 65°C for 5 min. Then reverse transcriptase buffer was added to a give a final concentration of 50 mM Tris-HCl (pH 8.3), 75 mM KCl, 3 mM MgCl_2_, 5 mM dithiothreitol (DTT). The final reaction mixture also contained 20 U RNasein (*Biolabs,* MO3145) and 40 U Superscript III. Temperature profiles used were 25°C for 5 min, 37°C for 10 min, 50°C for 80 min and the reaction was terminated at 72°C for 15 min. The samples were then stored at -80°C until further analysis.

For quantitative real time PCR analysis to determine Hsp27 RNA expression, we used a SYBR green method and actin as an internal control gene. The following primers were used for actin: Forward: ACAACGGCTCCGGCATGTGCAAAG; Reverse: CATTCCCACCATCACACCCTGGTGC. PCR reaction on mouse cells gave a 110 bp PCR product, which was then cloned into a TOPO TA holding vector (*Invitrogen*). Hsp27 forward primer: GCGGAAATACACGCTGCCC, Hsp27 reverse primer: TGGGATGGTGATCTCGTTGG, giving a 130 bp PCR product that was also cloned into a holding TOPO TA vector. Serial dilutions containing 100 to 100,000 copies of the plasmid were then used to determine the exact RNA copy numbers in our samples. Hsp27 mRNA copies were then normalized to actin to give comparable results between different mutants.

## The effects of mutant Hsp27 on neuronal survival

The human neuroblastoma-derived SH-SY5Y cell line (*ECACC*; Cat # 94030304) was used to establish the effects of Hsp27 mutants on neuronal survival using a Lactate Dehydrogenase (LDH) assay. Cells were grown in Dulbecco’s modified Eagle’s medium (DMEM-F12, *Gibco, Paisley, UK*) containing 15% fetal calf serum (FCS), 2 mM L-glutamine, 10 IU/ml penicillin, 100 μg/ml streptomycin (P/S) and 1% final volume non-essential amino acids. Cells were maintained at 37°C in a saturated humidity atmosphere of 95% air and 5% CO_2_. For the cell survival assay, the cells were plated at 5000 cells/well onto 96 well plates and 24 hours later the cells were transfected using Lipofectamine 2000 (*Invitrogen) and* incubated for 6 hours before the transfection medium was replaced with culture medium containing 10 μM all-trans retinoic acid (*Sigma)* in order to induce neuronal differentiation. At 3 days in vitro (DIV), some cultures were treated with H_2_O_2_ (100 µM) to mimic oxidative stress, or cytochalasin D (10 µM), that disrupts the actin cytoskeleton or colchicine (10 µM), that irreversibly binds to the tubulin cytoskeleton, causing alterations in the dynamic instability of the tubulin network. At 4 DIV, and 3 days following transfection, the effects of these treatments on cell survival were determined using an LDH assay (*Roche, Welwyn, UK*). Cell supernatants were incubated with LDH reagents for 40 min, then absorbance was measured at 490nm on a spectrophotometer. LDH values were normalized to protein levels and then compared between cells expressing WT Hsp27 and any of the Hsp27 mutations.

## Superoxide release measurements using MitoSOX

1. *Measurement of superoxide release by FACS analysis.*

Mouse primary motor neurons were plated onto 12 well plates (1,5x10^5^ cells/well) and transduced with mutant and WT Hsp27 24 hours after plating. At 5DIV, cells were incubated in culture media containing 0.5 µM MitoSOX™ for 25 min at 37°C. Cells were then quickly washed in HBSS and treated with 0.025% trypsin/EDTA (*Sigma* T3924) for 2 min, triturated and spun at 380g at 4°C for 3 min. The supernatant was removed and cells resuspended in 300 µl of HBSS.

MitoSOX was excited at 561 nm and the 588 nm emission intensity measured using the (FL3) channel on a FACS Calibur system (*BD Biosciences, San Jose, CA*). Data was collected in the SSC and FSC channels. Cell debris showing a low forward and side scatter, were filtered out from measurements. The FL1 channel was also excited using the 488 nm and green fluorescence was detected at 530 nm to identify cells expressing GFP, and thus containing human Hsp27. The green channel was thresholded using control uninfected cells and a series of cells infected using the lentiviral vectors expressing GFP, in order to only include GFP expressing, virally infected cells in the analysis. The red MitoSOX channel was also gated using cells infected with an empty viral construct. For each sample, a total of 5,000 gated cells were assessed and the percentage of cells positive for both MitoSOX and GFP calculated based on the total number of GFP positive cells detected under unstimulated conditions. The remaining cells in each tube were then treated with 20 µM antimycin A for 20 min and then re-analysed using the same FACS settings. The percentage of cells positive for both MitoSOX and GFP was calculated relative to the total number of GFP positive cells in each sample.

1. *Measurement of superoxide release by confocal microscopy*

MitoSOX intensity was also measured in primary motor neuron cultures at 5 DIV. Motor neurons plated on MatTek dishes (1x10^5^cells/dish) were incubated for 20-25 min in a working solution of MitoSOX (0.5 µM, as described above for the FACS analysis). Cells were then washed three times in RM and viewed at 37°C using the 488 nm and 561nm red lasers. Images were taken every 10 s under a 40x oil immersion objective. A baseline image was taken before addition of antimycin A (20 µM) to stress cells. Cells were then imaged for 25 min. Images and movies were viewed using the ZEN Lite software. Each cell body of GFP expressing neurons was delineated as regions of interest. Mean fluorescent intensities of each region of interest were then measured using the “Measurement” function in the ZEN software. In movies, the red (MitoSOX) fluorescent intensity measured in the last 10 frames (Maximum) was expressed as a multiple of the control intensity of the same cell at the beginning of the experiment.
