# Supplementary figures and images for: Mitochondrial deficits and abnormal mitochondrial retrograde axonal transport play a role in the pathogenesis of mutant Hsp27-induced Charcot Marie Tooth Disease
Source: Hum Mol Genet. 2017 Jun 8;26(17):3313–26. doi: 10.1093/hmg/ddx216 (PMC5808738; doi:10.1093/hmg/ddx216)

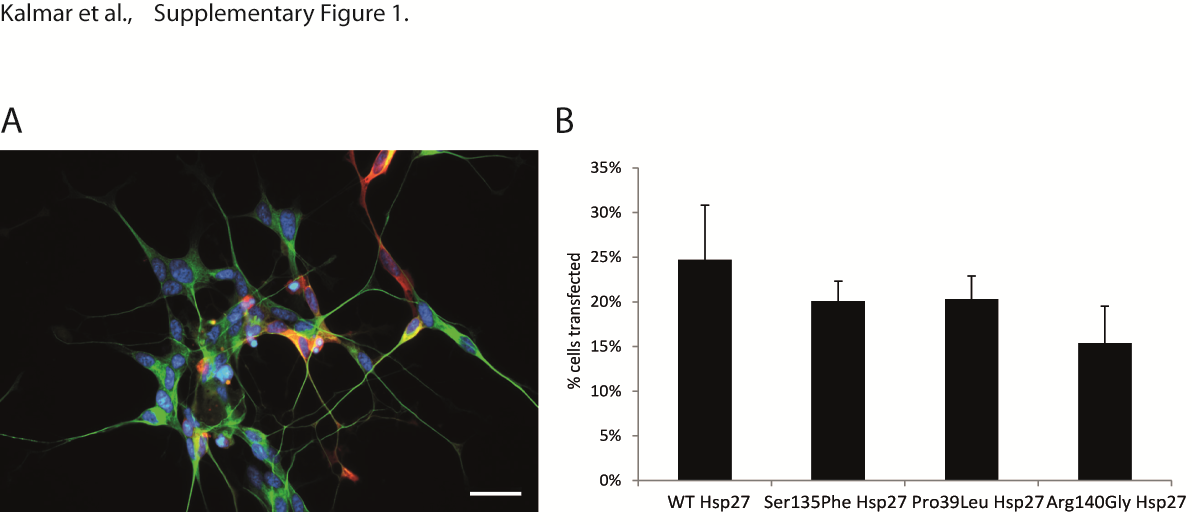

Supplement: Supplementary Figure 1 [file ddx216_kalmar_s_fig1low.png]

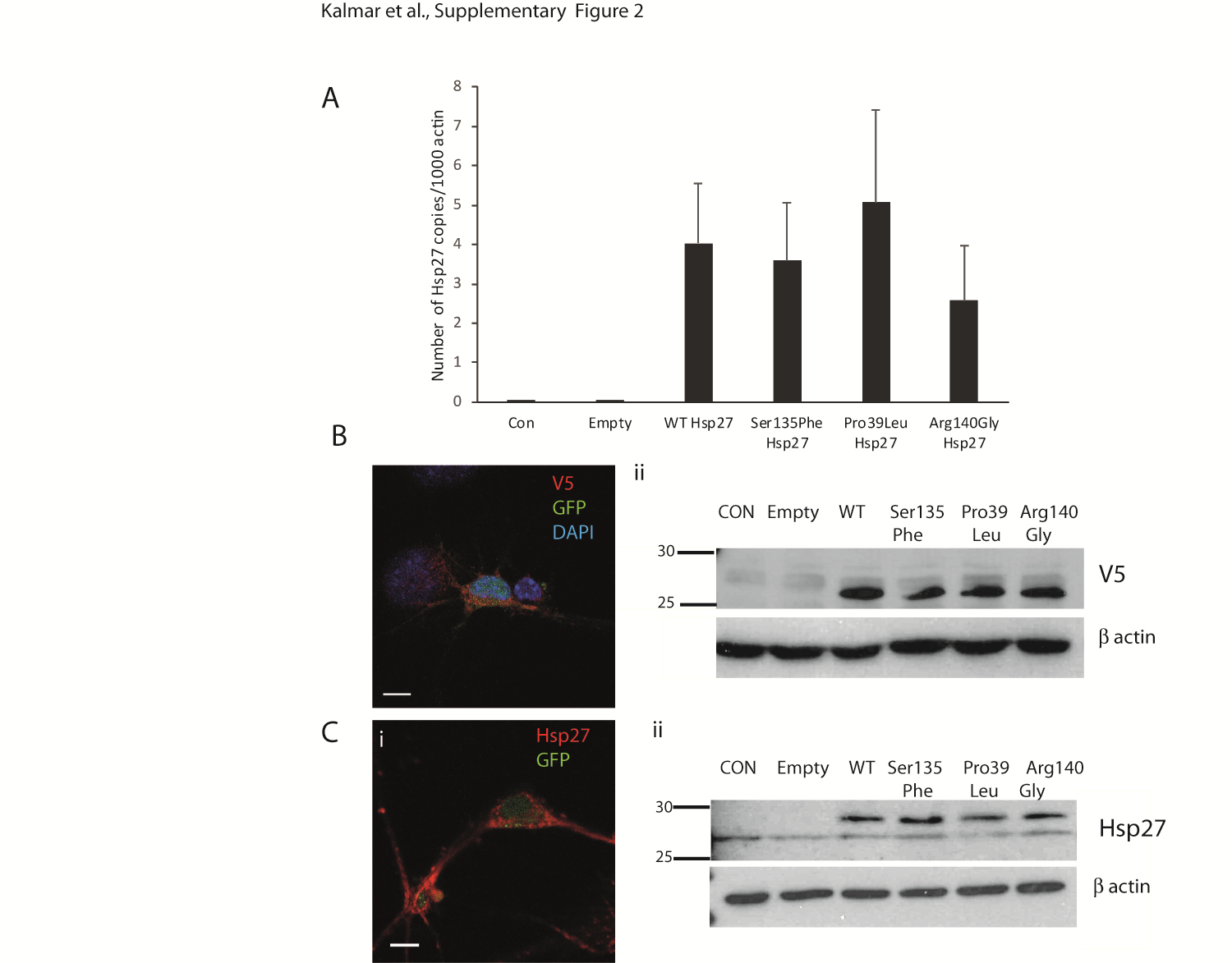

Supplement: Supplementary Figure 2 [file ddx216_kalmar_s_fig2low.png]

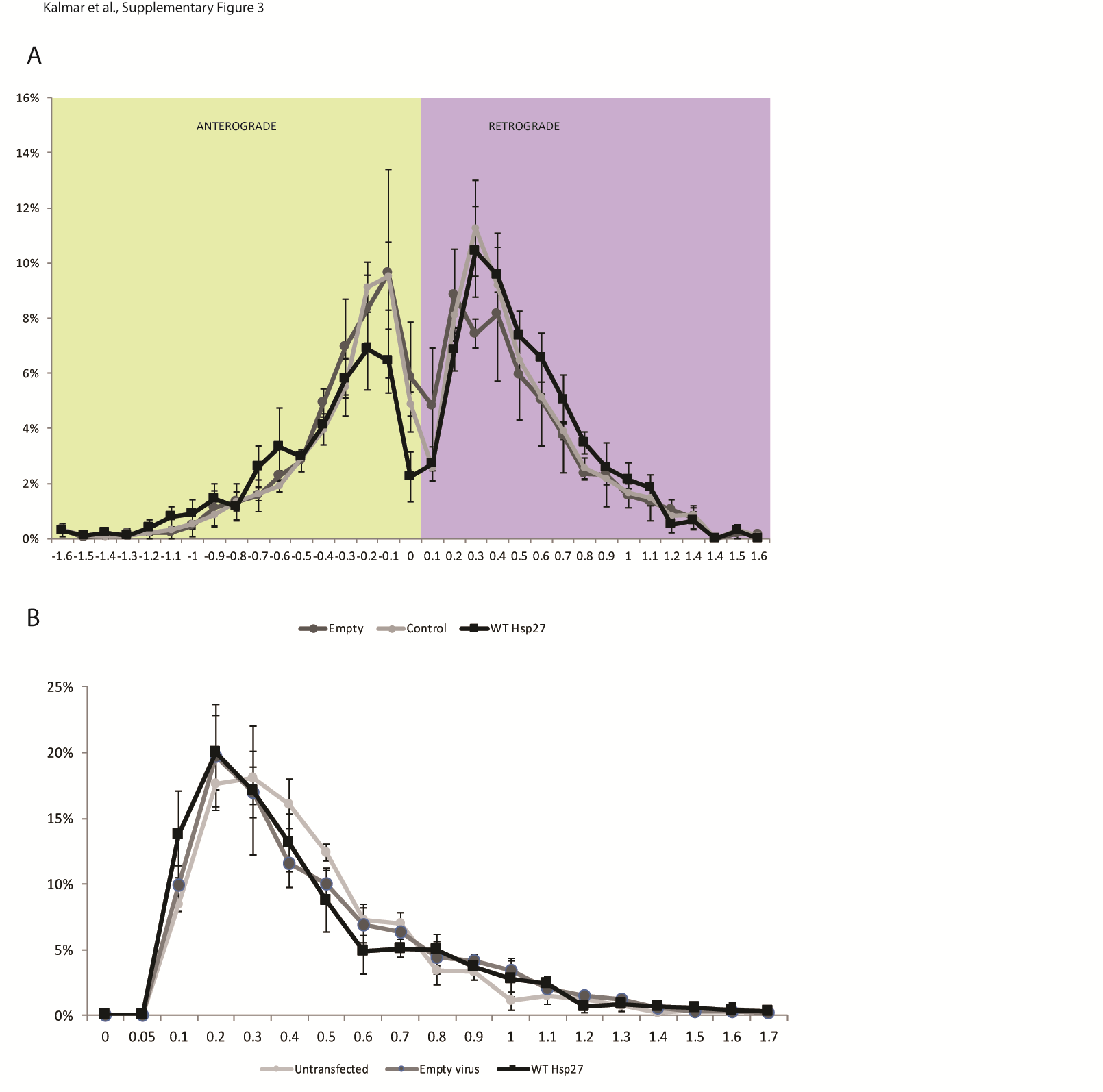

Supplement: Supplementary Figure 3 [file ddx216_kalmar_s_fig3low.png]
